# Bilateral Hemopneumothorax in COVID-19

**DOI:** 10.7759/cureus.10314

**Published:** 2020-09-08

**Authors:** Omer Salah, Mohannad Faisal, Israa Alshahwani, Abdelhaleem Elhiday

**Affiliations:** 1 Internal Medicine, Hamad Medical Corporation, Doha, QAT; 2 Epidemiology and Public Health, Hamad Medical Corporation, Doha, QAT

**Keywords:** pneumothorax, covid 19

## Abstract

A 50-year-old previously healthy male presented with fever and cough for seven days, positive for COVID-19, and was admitted to Hazm Meberik General Hospital and treated as a case of severe COVID-19 pneumonia. After improvement, he was transferred to a quarantine facility, and he later developed bilateral hemopneumothorax requiring bilateral chest tubes. High-resolution CT showed bilateral emphysematous bullous disease. Tuberculosis workup was negative, and alpha 1 anti-trypsin levels were normal. Repeated Chest X-ray showed improvement and chest tubes were removed. The patient was discharged with follow-up with the thoracic surgery clinic.

## Introduction

COVID 19, designated as severe acute respiratory syndrome coronavirus 2 (SARS-CoV-2), was identified in late 2019 as a cause of pneumonia.

Secondary spontaneous pneumothorax (SSP) is defined as pneumothorax that presents as a complication of underlying lung disease [1]. Nearly every lung disease can be complicated by SSP, although the most commonly associated diseases are chronic obstructive pulmonary disease and, in endemic areas, tuberculosis (TB). Other common causes include cystic fibrosis (CF), primary or metastatic lung malignancy, and necrotizing pneumonia [2].

Here, we present a case report of a patient who acquired COVID-19 pneumonia. Initially, he was having mild symptoms, but later he suddenly developed bilateral pneumohemothorax and bullous emphysematous disease, as revealed on high-resolution CT. These finding are not commonly known complications of COVID-19 and are mostly only presented in case reports.

We also reviewed some articles on the development of pneumothorax as a complication of COVID-19-associated pneumonia including tension pneumothorax, pneumomediastinum, and subcutaneous emphysema, in addition to case reports on cystic changes within the lungs as a sequel of COVID-19 infection.

## Case presentation

**Figure 1:**
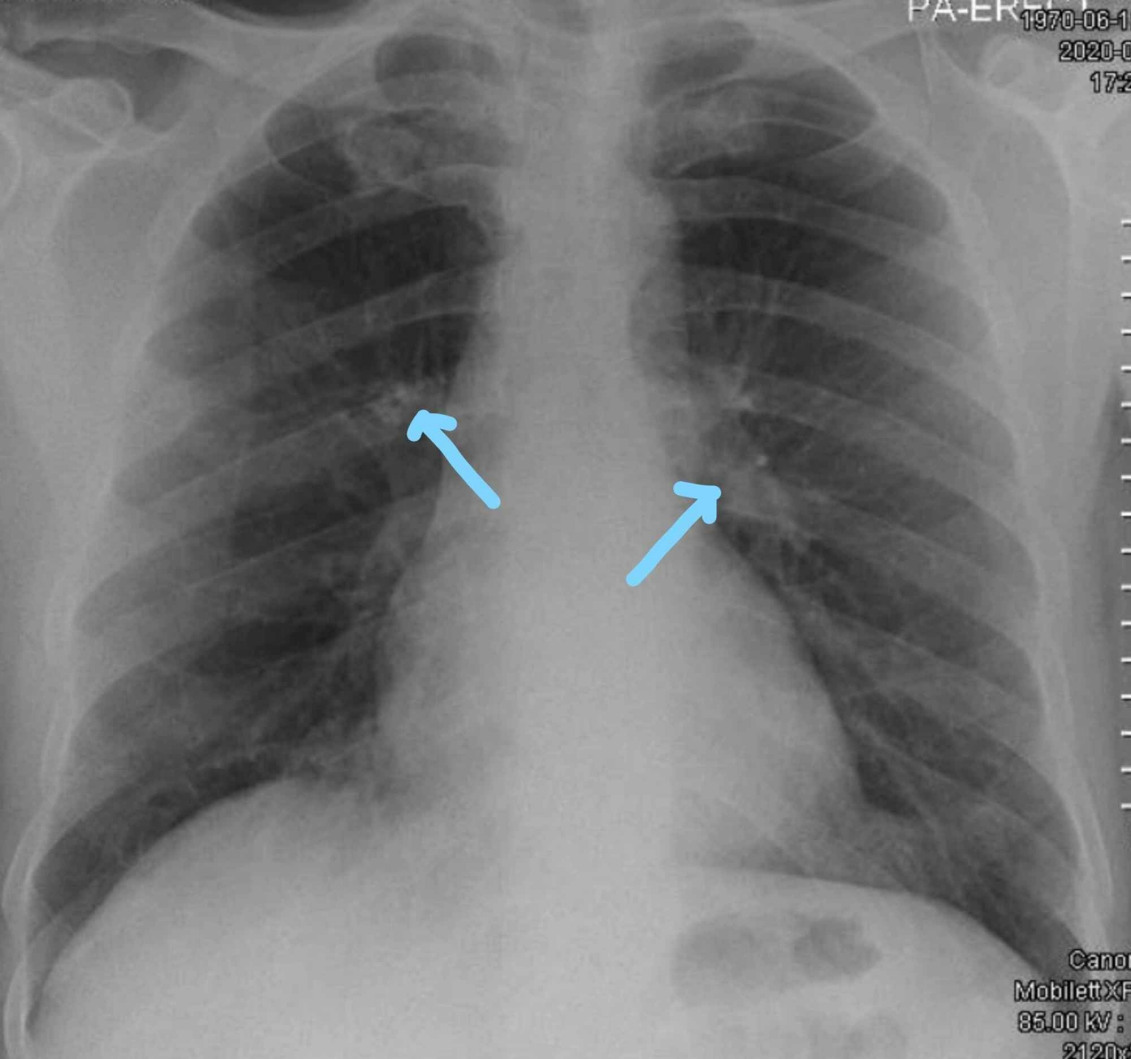
Chest X-ray on admission showing prominent bronchovascular markings and no infiltration

A 50-year-old previously healthy male presented to Al Wakrah Hospital with a seven-day history of fever and dry cough but did not experience any shortness of breath, chest pain, or palpitations. There was no history of smoking or alcohol use. He lives with three others in an apartment and works as a driver. Review of the system was unremarkable. Temperature was 37.1°C, pulse rate was 98 beats per minute, respiratory rate was 18 breaths/minute, and blood pressure (BP) was 145/85 mmHg. Chest examination showed equal breath sounds and no added sounds, and remaining of the examination was unremarkable. Initial chest X-ray (CXR) only showed prominent bronchovascular marking (Figure 1).

COVID-19 PCR (polymerase chain reaction) was positive. The patient was discharged with home isolation and was educated to come back if he developed worsening symptoms.

After six days, the patient developed high-grade fever, body aches, and shortness of breath that was increasing for the past three days. He was brought to the hospital through ambulance.

He was found to be febrile. Temperature was 39.1°C, respiratory rate was 22 breaths/minute, BP was 115/65 mmHg, pulse was 108 beats/minute, and SpO_2_ was 85% on room air. He was kept on 6L face mask and maintained a saturation of 95%. Examination showed equal breath sounds with bilateral crepitations.

**Figure 2:**
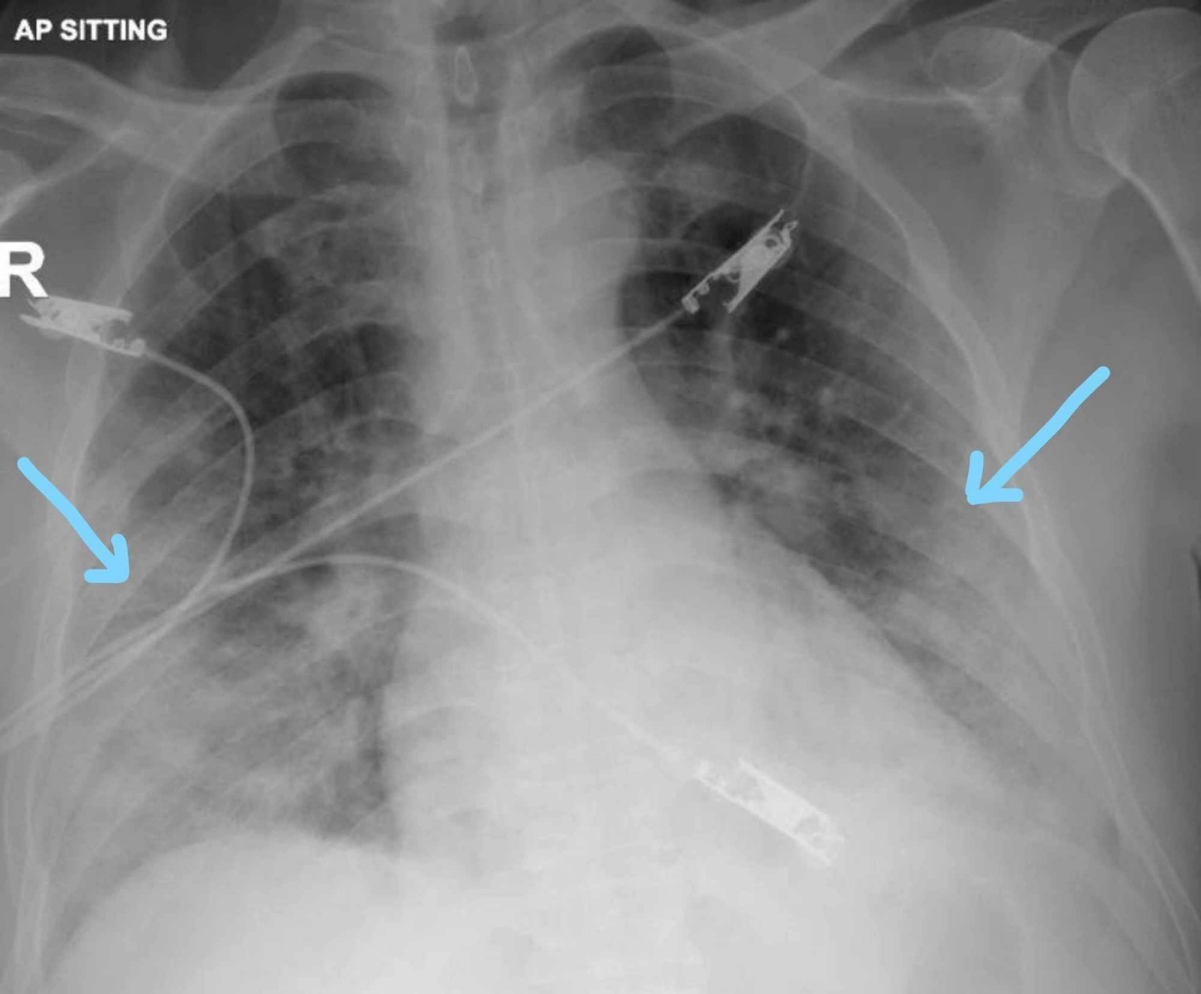
Bilateral infiltrates

On re-evaluation, he became more breathless and tachypneic (RR = 38) and saturation was 95% on a 15L non-rebreather mask. CXR showed multiple bilateral airspace infiltrates representing COVID-19 pneumonia (Figure 2). He was transferred to the intensive care unit (ICU) for continuity of care and was treated as a case of severe COVID pneumonia; Lab investigations are shown in Table 1.

**Table 1:** Basic labs investigation ALT, alanine amino transferase; ANC, absolute neutrophil count; APTT, activated partial thromboplastin time; Art, arterial; AST, aspartate amino transferase; BG, blood gas; CRP, C-reactive protein; G6PD, glucose 6 phosphate deficiency; Hct, hematocrit; Hgb, hemoglobin; INR, international normalized ratio; K, potassium; Na, sodium; POC, point of care; PO_^2^_, partial pressure of oxygen; PCO_2_, partial pressure of carbon dioxide; RBC, red blood cell count; WBC, white blood cell count

Group	Detail	Value, w/Units	Flags	Normal Range
POC BGs	pH Art-POC	7.491	Hi	7.350-7.450
	PO_2_ Art-POC	93 mmHg		83-108
	PCO_2_ Art-POC	36 mmHg		35-45
	Na Art-POC	130 mmol/L	Low	135-145
	K Art-POC	3.3 mmol/L	Low	3.5-5.0
	BG Hct Art-POC	42.0%		36.0-50.0
General hematology	WBC	10.2 x 10^3^/uL	Hi	4.0-10.0
	RBC	5.3 x 10^6^/uL		4.5-5.5
	Hgb	15.9 gm/dL		13.0-17.0
		8.9 x 10^3^/uL	Hi	2.0-7.0
	Lymphocyte	0.8 x 10^3^/uL	Low	1.0-3.0
Coagulation	Prothrombin time	13.8 seconds	Hi	9.4-12.5
	INR	1.2	NA	
	D-dimer	1.47 mg/L FEU	Hi	0.00-0.49
	APTT	36.0 seconds		25.1-36.5
Blood chemistry	Urea	4.1 mmol/L		2.8-8.1
	Creatinine	99 umol/L		62-106
	Total protein	75 gm/L		66-87
	Albumin level	27 gm/L	Low	35-52
	Alkaline phosphatase	197 U/L	Hi	40-129
	ALT	132 U/L	Hi	0-41
	AST	81 U/L	Hi	0-40
	G6PD screen	Normal		
	CRP	133.1 mg/L	Hi	0.0-5.0
Endocrinology	Ferritin	8,382.0 ug/L	Hi	30.0-553.0

The patient did not require intubation. He was started on hydroxychloroquine for 10 days, cefuroxime and azithromycin for 7 days, tocilizumab IV 400 mg single dose, and methylprednisolone 40 IV every 12 hours for 5 days. He also received convalescent plasma. The patient started to improve in terms of oxygen requirements and symptoms. After seven days of ICU admission, his care was de-escalated to the ward, and on 2L nasal cannula he was saturating well. Four days later, he was transferred to a quarantine facility.

**Figure 3:**
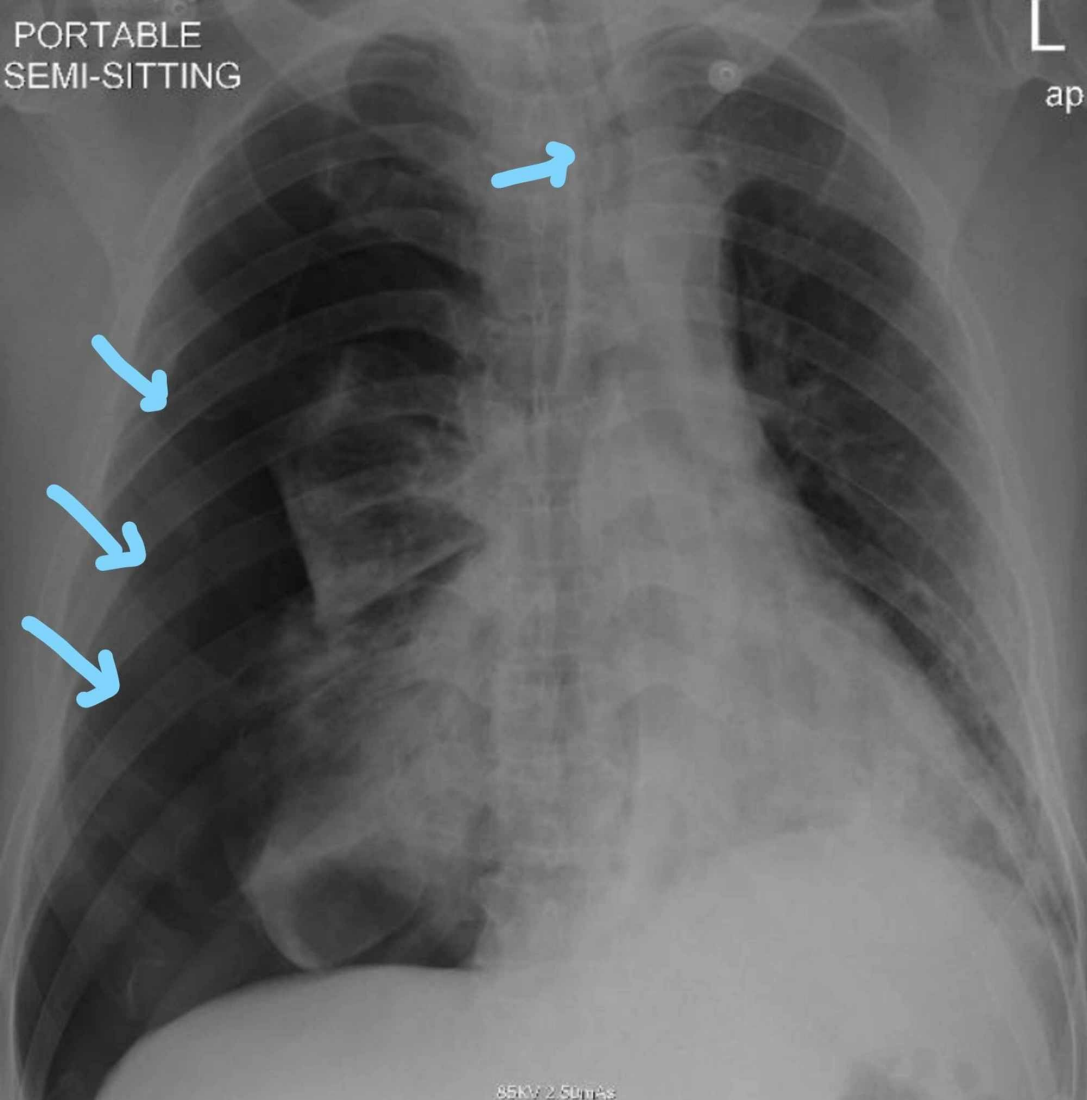
Right-sided pneumothorax with midline shift toward the left side

He was doing well until three days later where he started to deteriorate and developed shortness of breath and de-saturation. Repeat CXR showed right-sided pneumothorax with mild shift of the mediastinum to the left side (Figure 3).

**Figure 4:**
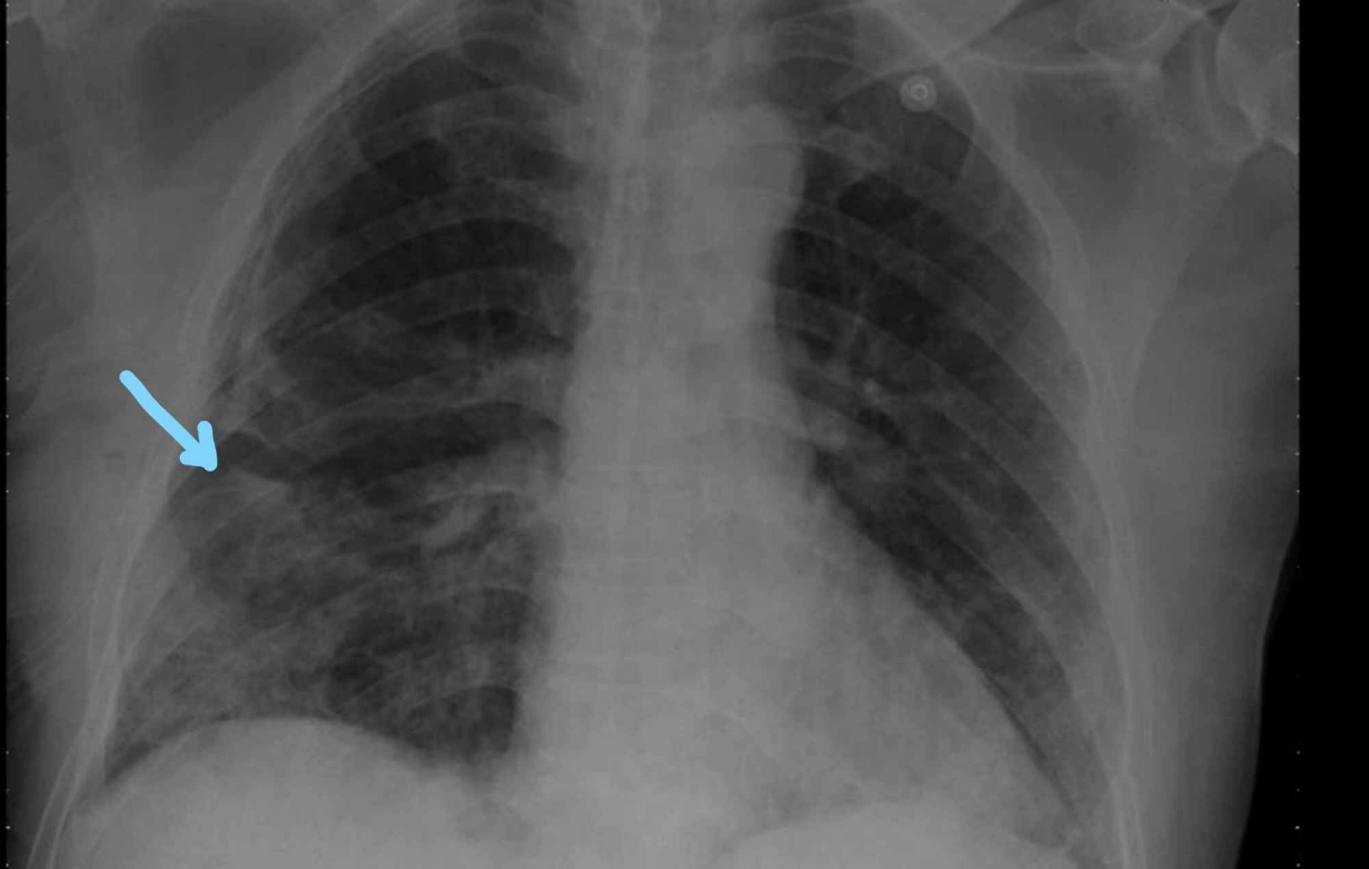
Right-sided chest tube insertion shows expansion of the right lung

Chest tube was inserted on the right side, and the right lung started to expand (Figure 4).

**Figure 5:**
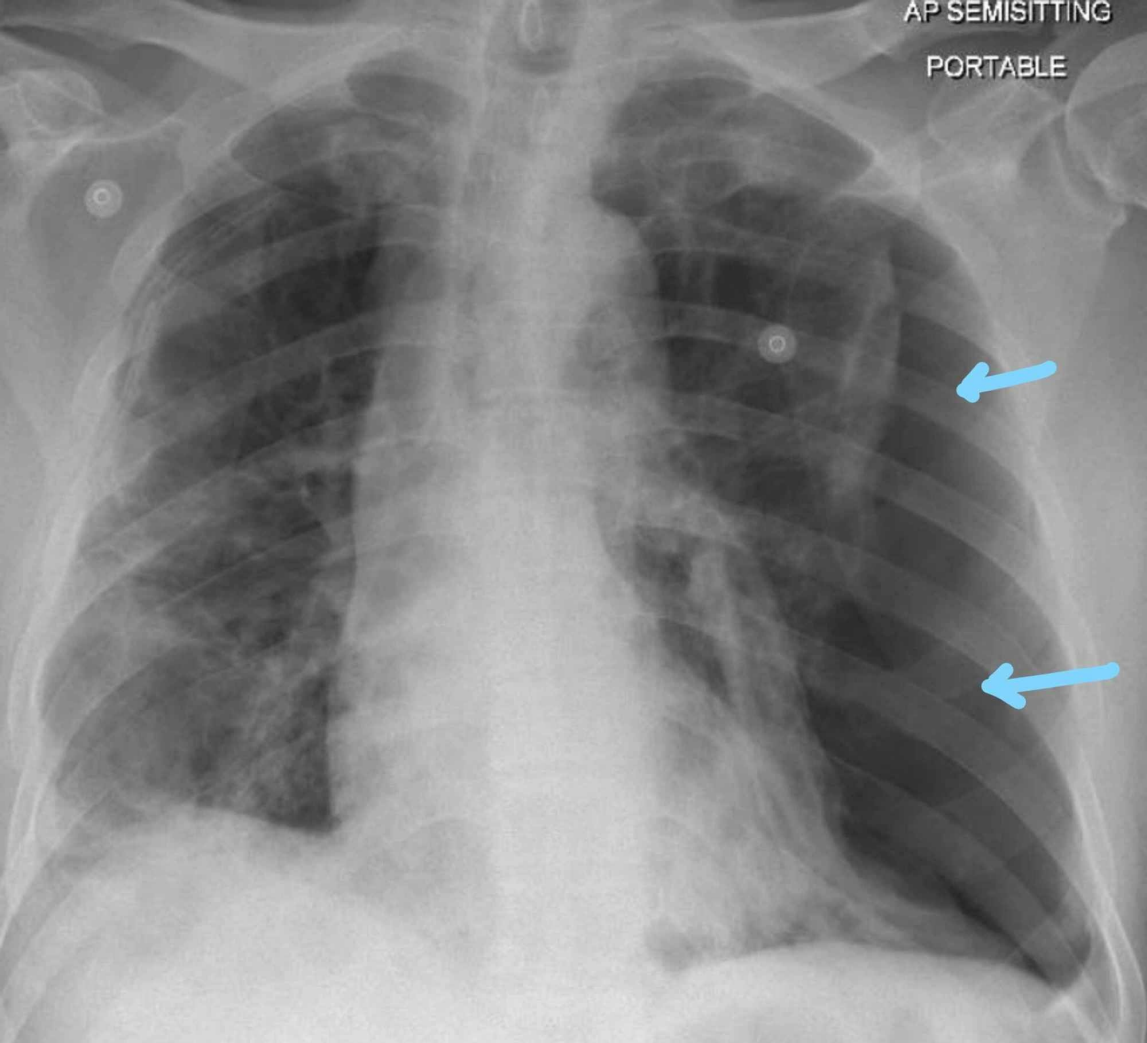
Large pneumothorax on the left side

The patient was transferred back to the hospital ICU. He was improving. Daily CXR was performed for follow-up, and three days later, repeat CXR showed newly developed left-sided pneumothorax (Figure 5).

Chest tube was inserted on the left side.

**Figure 6:**
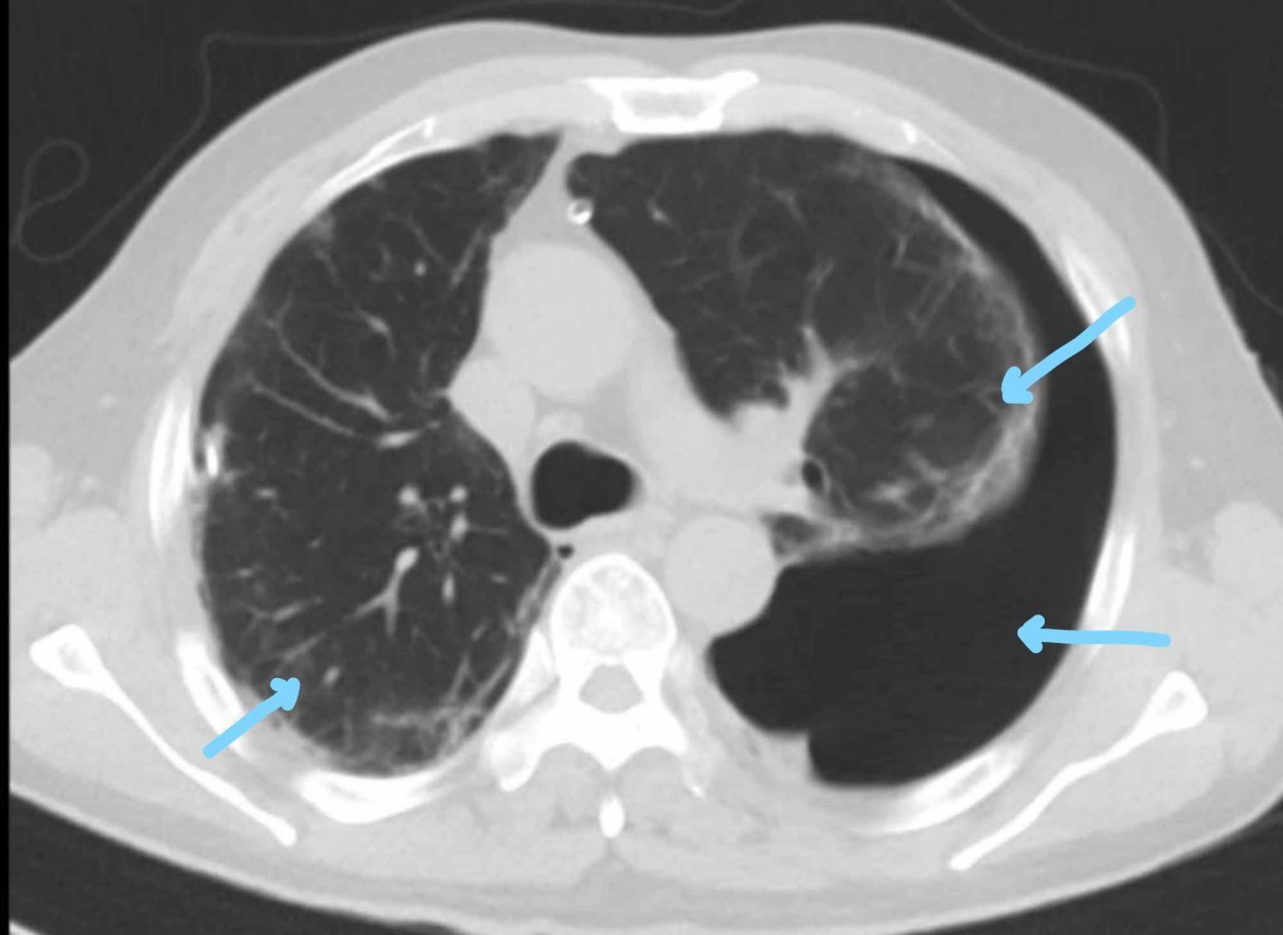
CT of the thorax demonstrating bilateral emphysematous bullous changes with pneumothorax

High-resolution CT (Figure 6) was performed, which showed bilateral emphysematous bullous complicated by pneumothorax due to rupture bullae with giant subpleural bullae in the left side measuring 19 x 7 cm and also a large one in the right side showing air-fluid level measuring 5.5 x 4.5 cm, indicating infected bullae versus lobulated hydropneumothorax. Also, there are bronchiectasis changes with multiple patchy areas of ground glass attenuation mainly in the mid and lower lobe, and diffuse centrilobular, peribronchial septal thickening and patchy nodular opacities were noted.

The right chest tube was removed as it stopped bubbling. He was saturating well on 2L nasal cannula. The patient was kept on salbutamol and tiotropium.

Two sets of acid-fast bacilli (AFB) sputum for TB, TB PCR, and culture were negative. Alpha 1 anti-trypsin level was normal.

**Figure 7:**
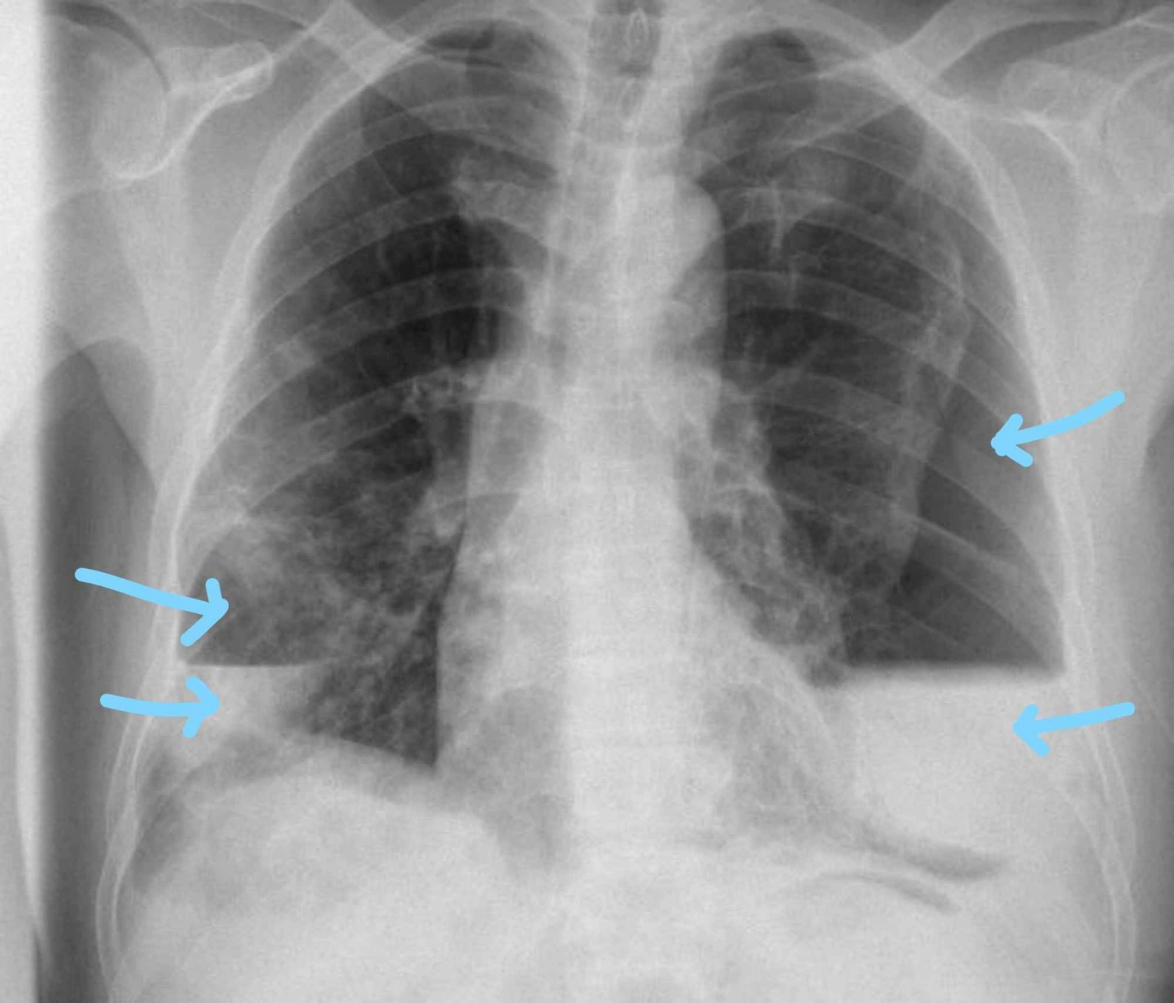
Bilateral air-fluid level mostly on the left lung

Follow-up CXR for the next five days showed no changes. On the sixth day, repeated CXR showed bilateral hydropneumothorax mostly on the left lung (Figure 7).

The patient was well, had no new symptoms, and was saturating well on 1L nasal cannula.

Another chest tube was inserted on the left side that drained 500 mL of blood along with air.

**Figure 8:**
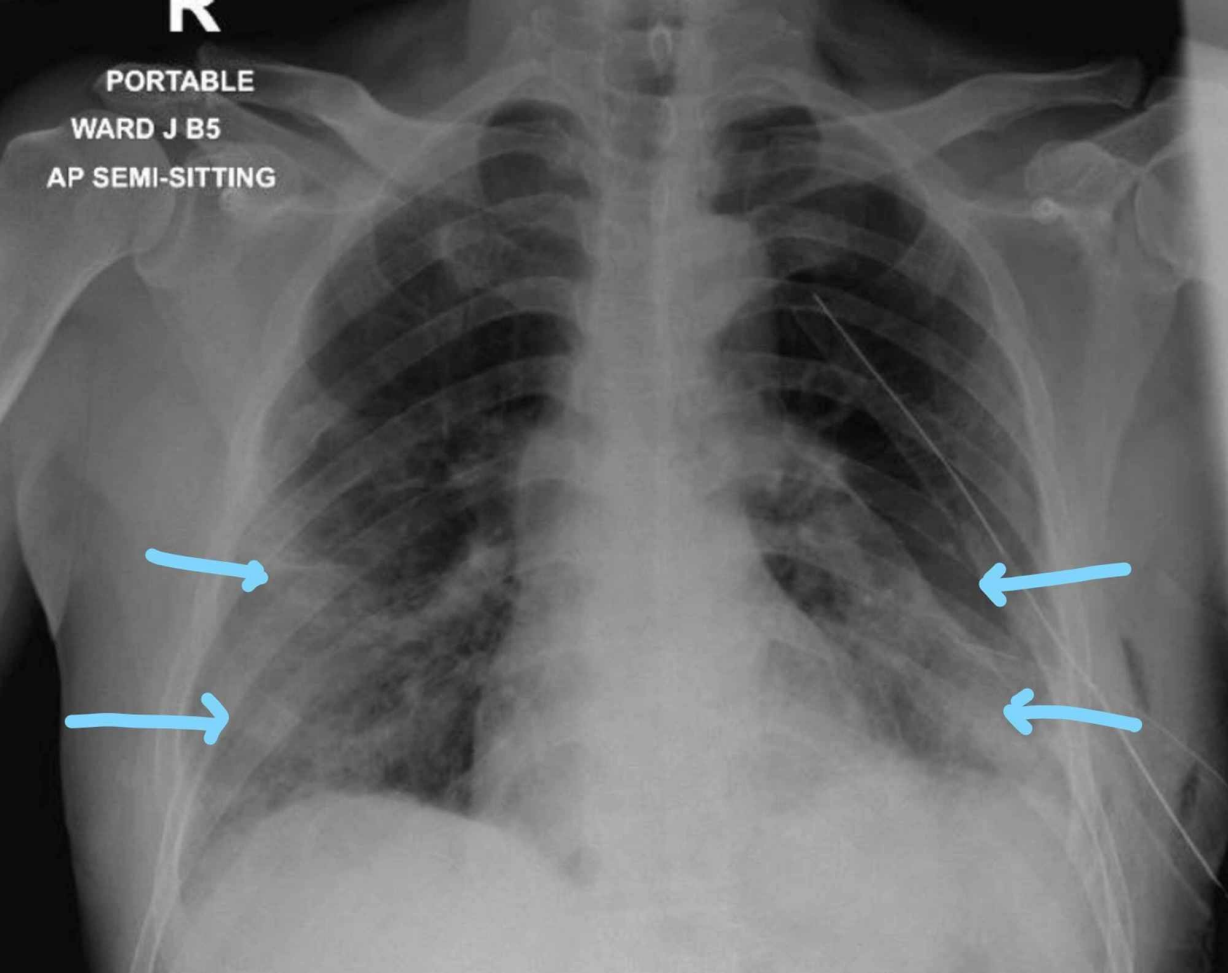
Resolution of bilateral hydropneumothorax

Repeat CXR after insertion of the chest tube on the next day showed resolution of hemothorax on the left side, and the right hemothorax resolved spontaneously (Figure 8).

**Figure 9:**
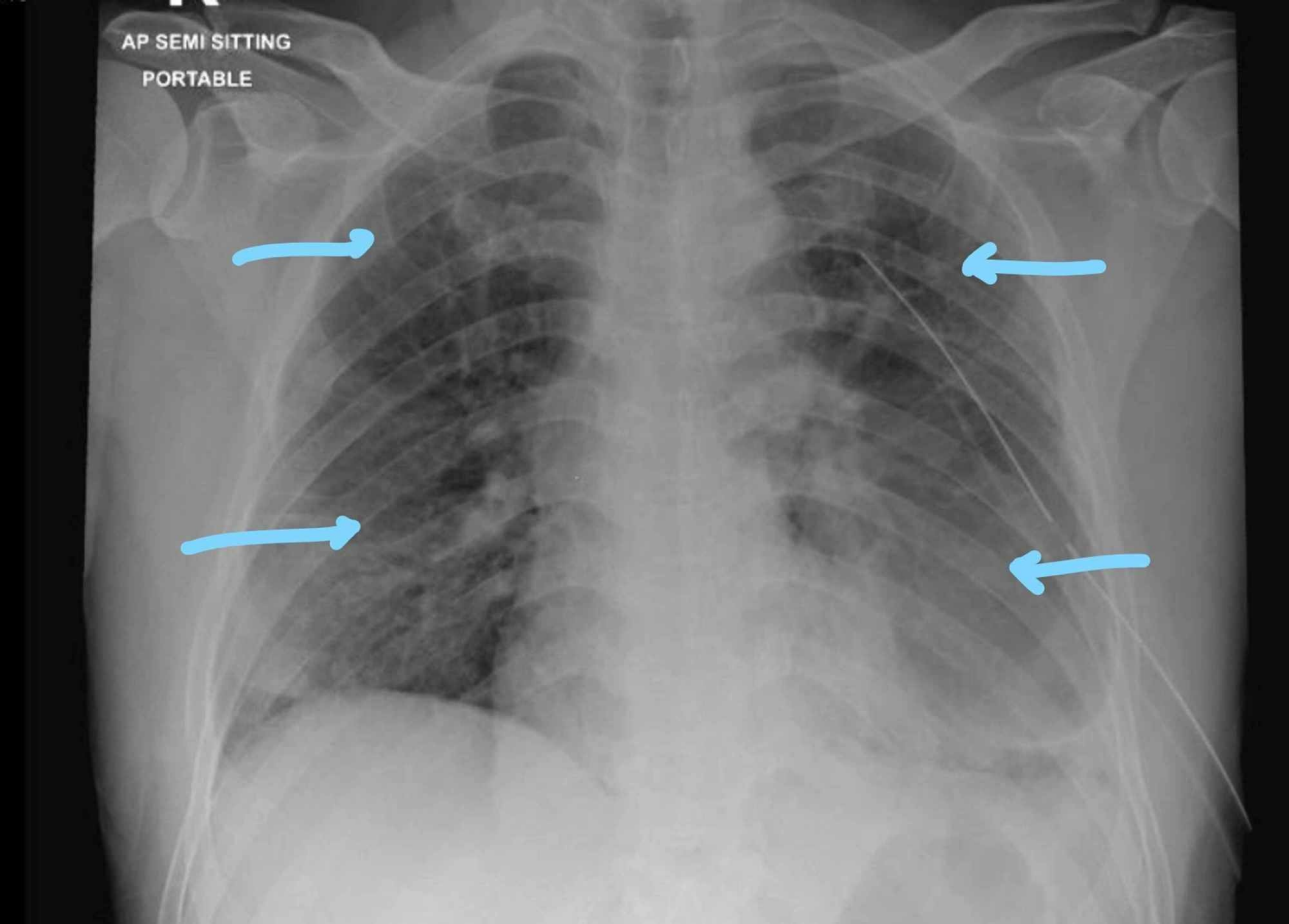
Repeat chest X-ray showing clamped chest tubes and inflated lungs

The small chest tube from the left side was removed, and the large bore tube was left. COVID 19 PCR was repeated and was negative. The chest tube stopped to bubble, not draining any fluid. The chest tube was clamped and CXR was repeated (Figure 9).

The chest tube was removed and the patient was monitored for four hours. He was feeling well and was discharged with follow-up with the thoracic surgeon.

## Discussion

In the described case, the patient with COVID-19 pneumonia had initially mild symptoms that later progressed requiring ICU admission but not requiring endotracheal intubation. As he improved, his hospital course got complicated with the development of bilateral pneumohemothorax with underlying bullae formation noticed in CT scan of the chest. He managed by placing bilateral chest tubes and was discharged in good health.

During his stay, he was worked up for other causes of the pneumothorax; two sets of TB AFB smear sputum, culture, and PCR were sent, which turned out negative. Alpha 1 anti-trypsin level was normal. His CT of the chest showed bilateral emphysematous bullous complicated by pneumothorax due to rupture bullae with giant subpleural bullae along with lobulated hydropneumothorax.

In view of the lack of previous history of underlying lung disease and the normal CXR on presentation along with the exclusion of some of the common causes of secondary pneumothorax, we think that COVID-19 pneumonia and its complication induced cystic changes within the lung and lead to spontaneous pneumothorax.

Pneumothorax has been reported as a complication of COVID-19 pneumonia and sometimes as a presenting feature reported by other case reports [3-9].

In our case, in addition to cases reported by Liu et al. [10], cystic changes within the lungs happened as a sequel of COVID-19 pneumonia, as evidenced by CT scan, and this case did not require intubation during the course of the illness [10]. We think that the COVID-19 infection and its sequel may have had a destructive effect on the lung parenchyma causing cystic changes and may have lead to subsequent pneumothorax, which need further reports and studies to be proven.

## Conclusions

COVID-19 could be a probable cause of spontaneous pneumothorax secondary to cystic changes and bullae formation within the lung parenchyma and should be kept in mind when evaluating patients with COVID-19 and shortness of breath, but we need further studies on the association between and pathophysiology of the occurrence of infection and pneumothorax.
